# (Naphthalene-1,4-di­yl)dimethyl dibenzoate

**DOI:** 10.1107/S1600536810008391

**Published:** 2010-03-17

**Authors:** Liang-You Xia

**Affiliations:** a Department of Chemistry, ZunYi Normal College, ZunYi 563002, People’s Republic of China

## Abstract

In the title compound, C_26_H_20_O_4_, the complete molecule is generated by a crystallographic 2-fold axis and the naphthalene ring system is planar within 0.05 (4) Å. The dihedral angles between the –COO plane, the benzene ring and naphthalene ring system are 12.83 (3) and 12.93 (1)°, respectively. The –COO plane and the benzene ring are almost coplanar, forming a dihedral angle of 2.59 (8)°.

## Related literature

For applications of related naphthalene derivatives, see: Fukuzumi *et al.* (1994 ▶); Madsen *et al.* (2002 ▶); Strey & Voss (1998 ▶); Tsukada *et al.* (1994 ▶).
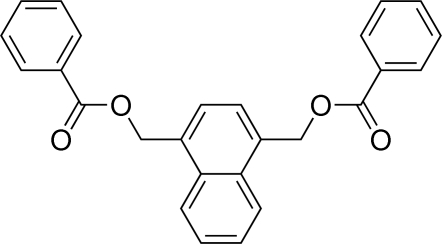


## Experimental

### 

#### Crystal data

C_26_H_20_O_4_
*M* *_r_* = 396.42
Orthorhombic, 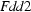

*a* = 3.9919 (6) Å
*b* = 60.385 (8) Å
*c* = 16.231 (2) Å
*V* = 3912.5 (9) Å^3^
*Z* = 8
Mo *K*α radiation
μ = 0.09 mm^−1^
*T* = 296 K
0.50 × 0.38 × 0.07 mm

#### Data collection

Bruker APEXII diffractometer
Absorption correction: multi-scan (*SADABS*; Sheldrick, 1996 ▶) *T*_min_ = 0.957, *T*_max_ = 0.994
6343 measured reflections
937 independent reflections
822 reflections with *I* > 2σ(*I*)
*R*_int_ = 0.027

#### Refinement

*R*[*F* ^2^ > 2σ(*F* ^2^)] = 0.033
*wR*(*F* ^2^) = 0.081
*S* = 1.08
937 reflections
136 parameters
1 restraint
H-atom parameters constrained
Δρ_max_ = 0.10 e Å^−3^
Δρ_min_ = −0.15 e Å^−3^

### 

Data collection: *APEX2* (Bruker, 2004 ▶); cell refinement: *SAINT* (Bruker, 2004 ▶); data reduction: *SAINT*; program(s) used to solve structure: *SHELXS97* (Sheldrick, 2008 ▶); program(s) used to refine structure: *SHELXL97* (Sheldrick, 2008 ▶); molecular graphics: *XP* in *SHELXTL* (Sheldrick, 2008 ▶); software used to prepare material for publication: *SHELXL97*.

## Supplementary Material

Crystal structure: contains datablocks global, I. DOI: 10.1107/S1600536810008391/om2316sup1.cif

Structure factors: contains datablocks I. DOI: 10.1107/S1600536810008391/om2316Isup2.hkl

Additional supplementary materials:  crystallographic information; 3D view; checkCIF report

## Figures and Tables

**Fig. 1.**
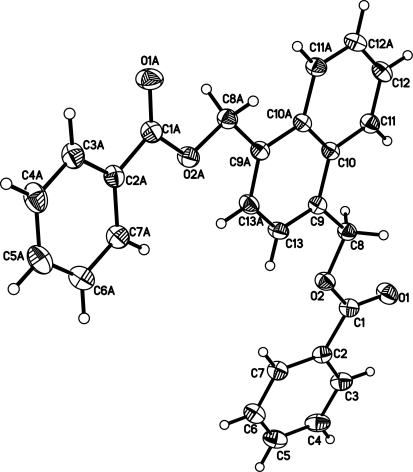
The molecular structure of the title compound showing 30% probability displacement ellipsoids and the atomic numbering. Symmetry code: A = 1.5 -x,0.5-y, z.

## supplementary crystallographic information

### Comment

Numerous 1,4-naphthalene derivatives have been synthesized and studied. 1,4-naphthalene derivatives are important intermediates for applications such as monomers in the preparation of polymers (Fukuzumi *et al.*, 1994; Tsukada *et al.*, 1994). The title compound has a 2-fold axis of symmetry passing through the long axis of napthalene (Fig. 1). The naphthalene ring system is planar within 0.05 (4)A°. The dihedral angles of the C1/O1/O2 plane, the C2—C7 ring and naphthalene ring are 12.83 (3)° and 12.93 (1)°, respectively. Molecules of the title compound are closely stacked with a repeat equal to the a-axial dimension.

### Experimental

The title compound was synthesized according to the reported procedure of Strey & Voss (1998) and Madsen *et al.* (2002). Colourless single crystals suitable for X-ray diffraction were obtained by recrystallization from ethyl acetate.

### Refinement

H atoms were positioned geometrically, with C-H = 0.93 and 0.97Å for aromatic and methylene H atoms, respectively, and constrained to ride on their parent atoms, Uiso(H)= 1.2Ueq(C). In the absence of significant anomalous scattering, Friedel opposites were merged (862 Friedel pairs).

### Crystal data

**Table:**

C_26_H_20_O_4_	*F*(000) = 1664
*M**_r_* = 396.42	*D*_x_ = 1.346 Mg m^−^^3^
Orthorhombic, *F**d**d*2	Mo *K*α radiation, λ = 0.71073 Å
Hall symbol: F 2 -2 d	Cell parameters from 1789 reflections
*a* = 3.9919 (6) Å	θ = 2.6–25.3°
*b* = 60.385 (8) Å	µ = 0.09 mm^−^^1^
*c* = 16.231 (2) Å	*T* = 296 K
*V* = 3912.5 (9) Å^3^	Block, colourless
*Z* = 8	0.50 × 0.38 × 0.07 mm

### Data collection

**Table:**

Bruker APEXII diffractometer	937 independent reflections
Radiation source: fine-focus sealed tube	822 reflections with *I* > 2σ(*I*)
graphite	*R*_int_ = 0.027
φ and ω scans	θ_max_ = 25.5°, θ_min_ = 2.6°
Absorption correction: multi-scan (*SADABS*; Sheldrick, 1996)	*h* = −4→4
*T*_min_ = 0.957, *T*_max_ = 0.994	*k* = −72→72
6343 measured reflections	*l* = −19→19

### Refinement

**Table:**

Refinement on *F*^2^	Primary atom site location: structure-invariant direct methods
Least-squares matrix: full	Secondary atom site location: difference Fourier map
*R*[*F*^2^ > 2σ(*F*^2^)] = 0.033	Hydrogen site location: inferred from neighbouring sites
*wR*(*F*^2^) = 0.081	H-atom parameters constrained
*S* = 1.08	*w* = 1/[σ^2^(*F*_o_^2^) + (0.0506*P*)^2^] where *P* = (*F*_o_^2^ + 2*F*_c_^2^)/3
937 reflections	(Δ/σ)_max_ < 0.001
136 parameters	Δρ_max_ = 0.10 e Å^−^^3^
1 restraint	Δρ_min_ = −0.15 e Å^−^^3^

### Special details

**Table:**

Geometry. All esds (except the esd in the dihedral angle between two l.s. planes) are estimated using the full covariance matrix. The cell esds are taken into account individually in the estimation of esds in distances, angles and torsion angles; correlations between esds in cell parameters are only used when they are defined by crystal symmetry. An approximate (isotropic) treatment of cell esds is used for estimating esds involving l.s. planes.
Refinement. Refinement of F^2^ against ALL reflections. The weighted R-factor wR and goodness of fit S are based on F^2^, conventional R-factors R are based on F, with F set to zero for negative F^2^. The threshold expression of F^2^ > 2sigma(F^2^) is used only for calculating R-factors(gt) etc. and is not relevant to the choice of reflections for refinement. R-factors based on F^2^ are statistically about twice as large as those based on F, and R- factors based on ALL data will be even larger.

### Fractional atomic coordinates and isotropic or equivalent isotropic displacement parameters (Å^2^)

**Table:**

	*x*	*y*	*z*	*U*_iso_*/*U*_eq_	
O1	0.1416 (5)	0.16848 (3)	0.65497 (12)	0.0680 (6)	
O2	0.4250 (4)	0.19760 (2)	0.60506 (10)	0.0507 (4)	
C1	0.2722 (6)	0.17767 (3)	0.59764 (15)	0.0467 (5)	
C2	0.2903 (6)	0.16879 (3)	0.51236 (15)	0.0452 (5)	
C3	0.1552 (6)	0.14804 (3)	0.49816 (16)	0.0556 (7)	
H3	0.0516	0.1404	0.5408	0.067*	
C4	0.1746 (8)	0.13876 (4)	0.42042 (17)	0.0652 (8)	
H4	0.0881	0.1247	0.4112	0.078*	
C5	0.3209 (7)	0.15021 (4)	0.35675 (18)	0.0655 (7)	
H5	0.3302	0.1440	0.3044	0.079*	
C6	0.4533 (7)	0.17084 (4)	0.37037 (17)	0.0611 (7)	
H6	0.5522	0.1786	0.3272	0.073*	
C7	0.4407 (7)	0.18018 (4)	0.44775 (16)	0.0522 (6)	
H7	0.5328	0.1941	0.4568	0.063*	
C8	0.4461 (6)	0.20608 (3)	0.68748 (14)	0.0455 (5)	
H8A	0.5784	0.1961	0.7213	0.055*	
H8B	0.2236	0.2071	0.7112	0.055*	
C9	0.6053 (5)	0.22851 (3)	0.68603 (13)	0.0413 (5)	
C10	0.6767 (5)	0.23923 (3)	0.76281 (14)	0.0391 (5)	
C11	0.6072 (6)	0.22913 (4)	0.83964 (14)	0.0474 (6)	
H11	0.5109	0.2151	0.8405	0.057*	
C12	0.6774 (7)	0.23935 (4)	0.91223 (14)	0.0537 (6)	
H12	0.6296	0.2323	0.9618	0.064*	
C13	0.6778 (6)	0.23930 (4)	0.61428 (14)	0.0468 (6)	
H13	0.6314	0.2324	0.5643	0.056*	

### Atomic displacement parameters (Å^2^)

**Table:**

	*U*^11^	*U*^22^	*U*^33^	*U*^12^	*U*^13^	*U*^23^
O1	0.0943 (15)	0.0554 (11)	0.0542 (11)	−0.0234 (10)	0.0100 (11)	0.0017 (9)
O2	0.0665 (11)	0.0417 (8)	0.0440 (9)	−0.0096 (7)	0.0002 (8)	−0.0032 (7)
C1	0.0565 (14)	0.0351 (10)	0.0486 (13)	−0.0024 (10)	−0.0037 (11)	0.0034 (11)
C2	0.0506 (13)	0.0390 (11)	0.0458 (13)	0.0011 (9)	−0.0074 (10)	0.0022 (10)
C3	0.0679 (17)	0.0407 (12)	0.0582 (17)	−0.0059 (11)	−0.0134 (13)	0.0024 (11)
C4	0.087 (2)	0.0445 (13)	0.0640 (18)	−0.0039 (13)	−0.0223 (15)	−0.0045 (13)
C5	0.084 (2)	0.0592 (15)	0.0538 (15)	0.0109 (14)	−0.0130 (14)	−0.0110 (14)
C6	0.0730 (18)	0.0615 (16)	0.0488 (15)	0.0047 (13)	0.0000 (13)	0.0010 (12)
C7	0.0627 (15)	0.0423 (11)	0.0517 (14)	−0.0017 (11)	−0.0027 (12)	0.0001 (11)
C8	0.0526 (13)	0.0420 (11)	0.0418 (13)	−0.0017 (10)	−0.0006 (11)	−0.0024 (11)
C9	0.0441 (13)	0.0398 (11)	0.0399 (13)	0.0027 (9)	−0.0010 (10)	0.0006 (10)
C10	0.0412 (11)	0.0398 (10)	0.0362 (11)	0.0054 (9)	0.0006 (10)	0.0003 (10)
C11	0.0558 (15)	0.0422 (11)	0.0442 (13)	−0.0007 (10)	0.0014 (12)	0.0053 (11)
C12	0.0667 (17)	0.0573 (14)	0.0371 (13)	0.0034 (11)	0.0039 (12)	0.0064 (10)
C13	0.0598 (14)	0.0445 (11)	0.0362 (12)	−0.0047 (10)	−0.0032 (11)	−0.0040 (10)

### Geometric parameters (Å, °)

**Table:**

O1—C1	1.202 (3)	C7—H7	0.9300
O2—C1	1.354 (3)	C8—C9	1.496 (3)
O2—C8	1.435 (3)	C8—H8A	0.9700
C1—C2	1.486 (3)	C8—H8B	0.9700
C2—C3	1.384 (3)	C9—C13	1.365 (3)
C2—C7	1.390 (3)	C9—C10	1.433 (3)
C3—C4	1.383 (4)	C10—C11	1.416 (3)
C3—H3	0.9300	C10—C10^i^	1.426 (4)
C4—C5	1.374 (4)	C11—C12	1.359 (3)
C4—H4	0.9300	C11—H11	0.9300
C5—C6	1.371 (4)	C12—C12^i^	1.410 (5)
C5—H5	0.9300	C12—H12	0.9300
C6—C7	1.378 (4)	C13—C13^i^	1.415 (4)
C6—H6	0.9300	C13—H13	0.9300
			
C1—O2—C8	115.25 (18)	O2—C8—C9	109.48 (18)
O1—C1—O2	122.4 (2)	O2—C8—H8A	109.8
O1—C1—C2	125.1 (2)	C9—C8—H8A	109.8
O2—C1—C2	112.43 (19)	O2—C8—H8B	109.8
C3—C2—C7	119.4 (2)	C9—C8—H8B	109.8
C3—C2—C1	117.6 (2)	H8A—C8—H8B	108.2
C7—C2—C1	123.03 (19)	C13—C9—C10	118.96 (18)
C4—C3—C2	119.8 (2)	C13—C9—C8	122.36 (19)
C4—C3—H3	120.1	C10—C9—C8	118.67 (18)
C2—C3—H3	120.1	C11—C10—C10^i^	118.26 (12)
C5—C4—C3	120.4 (2)	C11—C10—C9	122.17 (17)
C5—C4—H4	119.8	C10^i^—C10—C9	119.57 (11)
C3—C4—H4	119.8	C12—C11—C10	121.8 (2)
C6—C5—C4	120.0 (3)	C12—C11—H11	119.1
C6—C5—H5	120.0	C10—C11—H11	119.1
C4—C5—H5	120.0	C11—C12—C12^i^	119.92 (13)
C5—C6—C7	120.3 (3)	C11—C12—H12	120.0
C5—C6—H6	119.8	C12^i^—C12—H12	120.0
C7—C6—H6	119.8	C9—C13—C13^i^	121.47 (12)
C6—C7—C2	120.1 (2)	C9—C13—H13	119.3
C6—C7—H7	120.0	C13^i^—C13—H13	119.3
C2—C7—H7	120.0		
			
C8—O2—C1—O1	5.3 (3)	C1—C2—C7—C6	179.0 (2)
C8—O2—C1—C2	−174.01 (19)	C1—O2—C8—C9	−177.40 (18)
O1—C1—C2—C3	−2.0 (4)	O2—C8—C9—C13	6.7 (3)
O2—C1—C2—C3	177.2 (2)	O2—C8—C9—C10	−174.77 (18)
O1—C1—C2—C7	179.2 (3)	C13—C9—C10—C11	179.7 (2)
O2—C1—C2—C7	−1.6 (3)	C8—C9—C10—C11	1.2 (3)
C7—C2—C3—C4	0.8 (4)	C13—C9—C10—C10^i^	−0.4 (3)
C1—C2—C3—C4	−178.1 (2)	C8—C9—C10—C10^i^	−179.0 (2)
C2—C3—C4—C5	−1.4 (4)	C10^i^—C10—C11—C12	−0.2 (4)
C3—C4—C5—C6	1.0 (4)	C9—C10—C11—C12	179.7 (2)
C4—C5—C6—C7	0.0 (4)	C10—C11—C12—C12^i^	0.2 (5)
C5—C6—C7—C2	−0.6 (4)	C10—C9—C13—C13^i^	0.2 (4)
C3—C2—C7—C6	0.2 (4)	C8—C9—C13—C13^i^	178.7 (3)

Symmetry codes: (i) −*x*+3/2, −*y*+1/2, *z*.
